# Reliability and validity of a sedentary behavior questionnaire for South American pediatric population: SAYCARE study

**DOI:** 10.1186/s12874-019-0893-7

**Published:** 2020-01-10

**Authors:** Augusto César Ferreira De Moraes, Marcus Vinícius Nascimento-Ferreira, Claudia Lucia de Moraes Forjaz, Juan Carlos Aristizabal, Leticia Azzaretti, Walter Viana Nascimento Junior, Maria L. Miguel-Berges, Estela Skapino, Carlos Delgado, Luis A. Moreno, Heráclito Barbosa Carvalho

**Affiliations:** 1 Department of Epidemiology, School of Public Health, University of Sao Paulo, São Paulo, Brazil; 2 YCARE (Youth/Child cArdiovascular Risk and Environmental) Research Group, Faculdade de Medicina, Universidade de Sao Paulo, São Paulo, Brazil; 3 Growth, Exercise, NUtrition and Development (GENUD) Research Group, Instituto Agroalimentario de Aragon (IA2), Instituto de Investigacion Sanitaria Aragon (IIS Aragon), Centro de Investigacion Biomedica en Red de Fisiopatologıa de la Obesidad y Nutricion (CIBERObn), University of Zaragoza, Zaragoza, Spain; 4 Exercise Hemodynamic Laboratory, School of Physical Education and Sport, University of São Paulo, São Paulo, Brazil; 5 Grupo de Investigación en Fisiología y Bioquímica (PHYSIS), Universidad de Antioquia, Medellín, Colombia; 6 Escuela de Nutrición, Facultad de Medicina, Universidad de Buenos Aires, Buenos Aires, Argentina; 7 Metabolic Diseases, Exercise and Nutrition (DOMEN) Research Group, Federal University of Piaui, Teresina, Brazil; 8 Escuela de Nutrición, UdelaR, Montevideo, Uruguay; 9 Universidad Nacional Mayor de San Marcos (UNMSM), School of Medicine, Department of Pediatrics and Instituto Nacional de Salud del Niño (INSN), Neonatal Unit, Lima, Peru

**Keywords:** Youth, Methods, Sedentary behavior

## Abstract

**Background:**

Multicenter studies from Europe and the United States have developed specifically standardized questionnaires for assessing and comparing sedentary behavior, but they cannot be directly applied for South American countries. The aim of this study was to assess the reliability and validity of the South American Youth Cardiovascular and Environmental (SAYCARE) sedentary behavior questionnaire.

**Methods:**

Children and adolescents from seven South American cities were involved in the test-retest reliability (children: *n* = 55; adolescents: *n* = 106) and concurrent validity (children: *n* = 93; adolescents: *n* = 94) studies. The SAYCARE sedentary behavior questionnaire was administered twice with two-week interval and the behaviors were parent-reported for children and self-reported for adolescents. Questions included time spent watching television, using a computer, playing console games, passive playing (only in children) and studying (only in adolescents) over the past week. Accelerometer was used for at least 3 days, including at least one weekend day. We compared values of sedentary time, using accelerometers, by quartiles of reported sedentary behavior time and their sum.

**Results:**

The reliability of sedentary behavior time was moderate for children (rho ≥0.45 and k ≥ 0.40) and adolescents (rho ≥0.30). Comparisons between the questionnaire and accelerometer showed a low overall agreement, with the questionnaire systematically underreporting sedentary time in children (at least, − 332.6 ± 138.5 min/day) and adolescents (at least, − 399.7 ± 105.0 min/day).

**Conclusion:**

The SAYCARE sedentary behavior questionnaire has acceptable reliability in children and adolescents. However, the findings of current study indicate that SAYCARE questionnaire is not surrogate of total sedentary time.

## Background

The majority of epidemiologic studies addressing sedentary behavior uses questionnaire to assess sedentary activities [1]. Questionnaires are low cost and easy to administer, and can therefore be easily used in large-scale studies [2]. Despite the increased number of sedentary behavior questionnaires worldwide, [1, 3] few have been appropriately validated using psychometric criteria, [4] and those validated questionnaires were mainly developed to evaluate European and American pediatric population [5]. In this sense, authors of a recent systematic review argue that tools to measure sedentary behavior should be developed for Brazilian children and adolescents [6].

Although valid tools to evaluate sedentary behavior in South American pediatric population are scarce, [3–5] a recent systematic review showed only one questionnaire assessing psychometric properties in comparison with objective measure [5]. In this sense, in children and adolescents, no self-report or proxy-report sedentary behavior questionnaires are available that are both valid and reliable [5]. Thus, high-quality research required into the measurement properties of measurement instruments of sedentary behavior [5, 7].

Additionally, prior literature suggests that ethnic and socioeconomic differences are observed in sedentary behavior trends [8]. One solution to this gap is to use standardized data from a multicenter approach [9]. In Europe, multicenter approaches are largely used to prepare questionnaires for epidemiological researches, regarding their design, feasibility, reliability and validity [10–12]. Conversely, in South American there is no sedentary behavior questionnaire with its reliability and validity tested in pediatric population with multicenter approach. The rationale behind the standardization using multicenter approach is based on transcultural adaptation focused in the equivalence of meaning and semantic of the questions among different languages and countries [13]. For this reason, an observational, multicenter feasibility study was developed to investigate health behaviors in a South American pediatric population [14]. For this study, a cross-cultural sedentary behavior questionnaire was developed and we assessed the reliability and validity of this questionnaire in South American children and adolescents.

## Methods

### Study design

The current study is part of the South American Youth Cardiovascular and Environmental (SAYCARE) study. A pilot feasibility multicenter study which collected data from 237 children (3–10 years) and 258 adolescents (11–18 years), totalizing 495 participants. The inclusion criteria were: i) age ranging from 3 to 18 years old, ii) informed written consent signed by a parent (or legal guardian) or by adolescent participants before enrollment and iii) provide information about sex and age. The exclusion criteria were: i) pregnancy, ii) inability to complete the questionnaires and iii) refusing to sign the informed consent. A detailed information about SAYCARE study can be found elsewhere [14].

The reliability of the sedentary behavior questionnaire was assessed through test-retest stability and the concurrent validity was assessed by comparisons between questionnaire and accelerometer. For the reliability study, we collected data from Buenos Aires (Argentina), Lima (Peru), Medellin (Colombia), Montevideo (Uruguay), Santiago (Chile), and Sao Paulo and Teresina (Brazil), while, for validity study, we collected data from Lima, Medellin, Teresina and São Paulo. These cities were selected based on the presence of specialized research centers with experience in this area of research and a population of more than 500,000 inhabitants. We aimed to survey populations across different geographic areas.

The study occurred during the 2015 and 2016 academic years.

### Participants

We adopted assumptions of Nascimento-Ferreira et al. [15] for sample size estimation. The sample size for the reliability study was calculated using α-Cronbach = 0.75, α = 5% and β = 90%, [3, 16] while the parameters for the validity study were correlation coefficient = 0.40, α = 5% and β = 90% [3]. The estimated sample size was 136 participants for the reliability analysis, with a subsample of 65 participants randomly selected for the validity analysis. In each research center, at least 20 (10 children) and 16 participants (8 children) were selected for the reliability and validity analyses, respectively.

At the design level, participants from each center were equally distributed by sex (male and female) and school type (public and private). Accounting for potential sample losses, a 25% greater sample size was calculated, thereby involving a total of 170 participants in the reliability study and 81 participants in the validity study. In addition, anticipating a rejection rate of 40%, we invited 200 children and 200 adolescents for the reliability study and 110 children and 110 adolescents for the validity study, yielding a total of 400 participants to assess the reliability and validity of the SAYCARE sedentary behavior questionnaire.

### Data collection

To coordinate and ensure the consistency of our methodology, seven fieldwork teams (one from each city) participated in a general training workshop in order to obtain required qualifications for conducting fieldwork. Regarding data collection, we selected conveniently-located schools and sent formal invitations with detailed information about the study. Students who accepted the invitation to participate were required to complete an informed written consent signed by a parent (or legal guardian) and by adolescent participants prior to enrollment.

Participants were excluded if they were pregnant or if their questionnaires were incomplete; as well as, the absence of informed consent from parents, guardians, and/or the individual himself. Besides, participants who reported mobility issues were excluded from data analysis.

Participants in the reliability study completed the questionnaire at home twice, with 2-week interval between the first (Q1) and second administration (Q2). For children, the questionnaire was parent-reported and for adolescents was self-reported. For the validity study, participants wore an accelerometer (model GT3X, Manufacturing Technology Inc., Fort Walton Beach, FL, USA) during the week immediately before the administration of Q1. The accelerometer was used for seven consecutive days, for at least eight hours per day [11]. Participants were instructed to attach the accelerometer to their waist each morning immediately after waking up and to remove it before going to sleep or during any aquatic activity [11]. In addition, participants (adolescents) or their parents/guardian (children) completed a record documenting the periods of accelerometer removal/replacement.

### SAYCARE questionnaires

The SAYCARE sedentary behavior questionnaire was developed from questionnaires used in European multicenter studies [10, 11]. The questions were designed to assess screen time [3, 17]. In addition, questions about behaviors related to health outcomes in Brazilian pediatric population were included [6].

Based on varying cultural backgrounds, a cross-cultural questionnaire was adapted for all research centers (cities) in two versions/languages, Portuguese (for Brazilian cities) and Spanish (other cities), following the instructions for a tool design and development [1, 6]. In this sense, we performed a cross-cultural adaptation (with translation and back translation) from European Spanish to Brazilian Portuguese and South American Spanish languages in two steps i) meaning and ii) semantic equivalence which might be directly related with questions comprehension [13].

Sedentary behavior time was assessed in the previous week through questions about time expended in the following sedentary activities: watching television, using a computer, studying, and playing console games for adolescents; in children, time spent studying was replaced with passive play (e.g., playing with a toy or doll, painting). A total of 8 questions each for children and adolescents were asked.

The time spent in sedentary behavior was measured as the sum of all time reported (min/day) performing sedentary activities, separately for weekdays and weekend days. The total sedentary behavior time, for complete week, was calculated as follows: [11].

$$ \frac{\Big[\left(\mathrm{sedentary}\ \mathrm{behavior}\ \mathrm{on}\ \mathrm{weekdays}\ \mathrm{x}\ 5\right)+\left(\mathrm{sedentary}\ \mathrm{behavior}\ \mathrm{on}\ \mathrm{weekend}\ \mathrm{days}\ \mathrm{x}\ 2\right)}{7} $$

In addition, participants were classified as achieving or not achieving the recommended limit of less than 120 min/day (2 h/day) of sedentary behavior [18]. Demographic and socioeconomic information were also collected.

### Accelerometers

The accelerometers were configured for a frequency of 30 Hz, an epoch of 5 s and one axis [11, 19]. Device data were only analyzed when valid measures were obtained for at least three days, including two weekdays and one weekend day [11]. Data from the days when the monitor was delivered and returned were excluded from the analysis. In addition, periods with 0 (zero) counts per minute (cpm) for more than 20 min were excluded as periods of non-use, [11] whereas periods with more than 20,000 cpm were excluded as a potential malfunction. Sedentary activity was defined as activity lower than 100 cpm [20]. We standardized the sedentary time each day correcting by accelerometer wearing time [21]. In this sense, sedentary time was expressed amount of time accumulated below 100 cpm during periods when the accelerometer was worn based on proportion of monitor-wearing time [11, 21]. Sedentary time (min/day) was assessed for weekdays and weekend days. Total sedentary time (for complete week) was calculated as follows: [11]

$$ \frac{\left(\mathrm{sedentary}\ \mathrm{time}\ \mathrm{on}\ \mathrm{weekdays}\ \mathrm{x}\ 5\right)+\left(\ \mathrm{sedentary}\ \mathrm{time}\ \mathrm{on}\ \mathrm{weekend}\ \mathrm{days}\ \mathrm{x}\ 2\right)}{7} $$

In addition, participants were classified as achieving or not achieving the recommended limit of less than 120 min/day (2 h/day) of sedentary time [18].

### Statistical analysis

Analyses were conducted in Stata 14 software (StataCorp, College Station, TX, USA). Shapiro-Wilk was used to determine sample normality. Descriptive analyses of demographic and socioeconomic variables are presented. In the sensitivity analyses, we assessed differences between categorical variables using the Chi-square goodness-of-fit test. We measured the test–retest reliability, agreement between Q1 and Q2, using a Spearman correlation coefficient (rho) for continuous variables and Cohen’s kappa (k) agreement for categorical variables. We assessed unweighted k-coefficients to identify participants who followed the recommendations and weighted (quadratic) k-coefficient to compare objectively measured sedentary time by groups (quartiles) of self-reported sedentary behavior time.

To assess the validity, for agreement between Q1 and accelerometer, we used Spearman correlation coefficient and kappa agreement. A moderate Spearman correlation (rho ≥0.30) [22, 23] and kappa coefficient (k ≥ 0.40) [24] were considered acceptable agreements. Additionally, we used Bland-Altman analysis to assess disagreement between Q1 and accelerometer. The range of agreement was defined as the mean bias ±1.96 standard deviations (and 95% limits of agreement, LOA) and heteroscedasticity between the measures was checked using the Pitman (Trend) test [25]. Heteroscedasticity was examined to verify whether the absolute intermethods difference (bias) was associated with the magnitude of the mean of sedentary behavior measured (ie, intermethods mean) [25]. We considered a *P* value < 0.05 statistically significant.

## Results

Altogether 495 participants met the general SAYCARE inclusion criteria (age range 3–18 years, signed an informed written consent and provided information about sex and age). Among these, 415 (at least 83.8%) completed sedentary behavior Q1. Thus, we have found incomplete (or missing in the Q1) information about sedentary behavior in 16.2% of the valid sample. For the reliability study, data from 161 participants were analyzed (Q2 response rate, 38.8%). For the validity analysis, data from 187 participants were analyzed (accelerometer response rate, 45.1%), most valid data (from the Q1 and accelerometer) came from the Brazilian cities (Table 1). Moreover, in the reliability study, our sample was composed by 9.3% of the participants from Buenos Aires, 23.0% from Lima, 36.6% from Medellin, 6.8% from Montevideo, 5.0% from Santiago, 17.4% from São Paulo and 1.9% from Teresina; while in the validity study, our sample was composed by 9.1% of the participants from Lima; 10.7% from Medellin; 47.6% from São Paulo and 32.6% from Teresina. The complete distribution of participants is shown in Additional file 1: Table S1. For total days, test-retest reliability of sedentary behavior time (min/day) for parent-report in children yielded a rho of 0.70; whereas, yielded a rho of 0.50 for self-report in adolescents. In addition, the reliability of quartile agreement and percentage of parent-report for children who followed the recommendations was k ≥ 0.40 (Table 2). The questionnaire showed low concurrent validity and bias between the questionnaire and the accelerometer was negative. Further analyses showed a wide LOA (Table 3).

**Table 1:** Distribution of sample in terms of demographic and socioeconomic variables of the SAYCARE general sample; and reliability and validity study samples

Children	SAYCARE sample (Q1, *N* = 200)	Reliability study sample (Q2, *N* = 55)	Validity study sample (Accelerometry data, *N* = 93)	P1	P2
	%	%	%		
Sex				0.150	0.746
Male	47.8	60.0	50.0		
Female	52.2	40.0	50.0		
Age				**< 0.001**	**0.033**
3–5 years	55.5	34.5	43.9		
6–10 years	44.5	65.5	56.1		
Maternal education level				0.919	0.195
Incomplete high school	20.9	15.6	13.6		
High school	13.3	12.5	20.4		
Technical education	10.8	12.5	4.5		
University degree	55.1	59.4	61.4		
School type				**< 0.001**	**< 0.001**
Public	61.5	21.8	34.1		
Private	38.5	78.2	65.9		
Adolescents	SAYCARE sample (Q1, *N* = 215)	Reliability study sample (Q2, *N* = 106)	Validity study sample (Accelerometry data, *N* = 94)	P1	P2
	%	%	%		
Sex					
Male	50.0	39.4	50.0	0.643	0.99
Female	50.0	60.6	50.0		
Age					
11–14 years	51.9	46.2	40.3	0.115	0.115
15–18 years	48.1	53.8	59.6		
Maternal education level					
Incomplete high school	15.6	15.8	11.6	**0.019**	**0.020**
High school	23.4	23.7	16.3		
Technical education	15.6	15.8	4.6		
University degree	45.4	44.7	67.4		
School type					
Public	30.8	36.8	29.7	**< 0.001**	0.847
Private	69.2	63.2	70.3		

Q1: Questionnaire first application; Q2: Questionnaire second application

P1: Proportion comparisons between Q1 and Q2 sample distributions

P2: Proportion comparisons between Q1 and participants with accelerometry data sample distributions

Significant values (*p* ≤ 0.05) are in **bold**

**Table 2:** Reliability analysis (test–retest) of the SAYCARE sedentary behavior questionnaire

**Children (N=55)**	**Week days**				**Weekend days**				**Total days**			
	**Q1**	**Q2**	**rho**	**k**^**a**^	**Q1**	**Q2**	**rho**	**k**^**a**^	**Q1**	**Q2**	**rho**	**k**^**a**^
Total SB time (min/day)	66.1 (41.0 to 96.0)	58.5 (46.3 to 112.6)	0.45*	0.55**	200.0 (135.0 to 285.0)	180.0 (120.0 to 240.0)	0.51**	0.40*	110.5 (74.0 to 154.3)	120.0 (75.0 to 142.1)	0.70**	0.53**
Meeting ≤120min/day (%)	85.8	75.9		**0.52**^b,^ *	15.8	15.9		**0.83**^b,^ *	42.2	47.7		**0.63**^b,^ *
**Adolescents (n=106)**	**Week days**				**Weekend days**				**Total days**			
	**Q1**	**Q2**	**rho**	**k**^**a**^	**Q1**	**Q2**	**rho**	**k**^**a**^	**Q1**	**Q2**	**rho**	**k**^**a**^
Total SB time (min/day)	102.0 (63.4 to 165.9)	54.7 (18.7 to 96.7)	0.39*	0.13	180.0 (96.4 to 260.0)	90.1 (18.8 to 186.0)	0.34**	0.27**	131.1 (97.1 to 178.2)	74.9 (27.4 to 127.0)	0.50**	0.28**
Meeting ≤120min/day (%)	55.4	84.6		0.15^b,^ *	31.8	84.5		0.08^b,^ *	40.1	73.1		0.17^b,^ *

Values are median (25^th^–75^th^ percentile); *k* kappa agreement, *Q1* Questionnaire first application, *Q2* Questionnaire second application, *rho* Spearman correlation coefficient, *SB* sedentary behavior

Moderate (or above) values of spearman correlation (rho ≥ 0.30) and kappa agreement (k ≥ 0.40) are in bold

^a^weighted (quadratic) Cohen’s kappa-coefficient for quartiles comparison;

^b^Cohen’s kappa coefficient for binary data;

*p ≤0.05

**p ≤0.01

**Table 3:** Validity analysis of the SAYCARE sedentary behavior questionnaire

**Children (N=93)**	**Q1**	**Accelerometry data**	**Bland-Altman Analysis**			**rho**	**k**^**d**^
	**Median (25**^**th**^**–75**^**th**^ **percentile)**	**Median (25**^**th**^**–75**^**th**^ **percentile)**	**Bias**^**a**^	**95%LOA**^**b**^	**Trend**^**c**^		
Week days (min/day)	66.1 (41.0 to 96.0)	522.4 (491.7 to 547.8)	-459.5 ± 100.9	-661.4 to -257.6	- 0.57**	- 0.10	0.14
Weekend days (min/day)	200.0 (135.0 to 285.0)	546.6 (502.4 to 598.2)	-332.6 ± 138.5	-609.6 to -55.6	0.20	0.40**	0.04
Total days (min/day)	110.5 (74.0-154.3)	530.4 (497.2 to 560.2)	-420.2 ± 100.3	-620.9 to -219.5	-0.45**	0.07	0.03
**Adolescents (N=94)**	**Q1**	**Accelerometry data**	**Bland-Altman Analysis**			**rho**	**k**^**d**^
	**Median (25**^**th**^**–75**^**th**^ **percentile)**	**Median (25**^**th**^**–75**^**th**^ **percentile)**	**Bias**^**a**^	**95%LOA**^**b**^	**Trend**^**c**^		
Week days (min/day)	102.0 (63.4 to 165.9)	570.3 (545.6 to 599.7)	-449.3 ± 87.0	-623.3 to -275.3	- 0.16	-0.26	-0.11
Weekend days (min/day)	180.0 (96.4 to 260.0)	599.2 (568.9 to 627.6)	-399.7 ± 105.0	-609.6 to -189.7	0.66**	0.21	0.36**
Total days (min/day)	131.1 (97.1 to 178.2)	582.2 (554.3 to 609.1)	-435.1 ± 66.3	-566.6 to -302.6	0.08	0.06	0.05

Values are median (25^th^–75^th^ percentile). *Q1* Questionnaire first application, *rho* Spearman correlation coefficient

Moderate (or above) values of spearman correlation (rho ≥ 0.30) and kappa agreement (k ≥ 0.40) are in bold

^a^Bias: average difference between methods (Q1 and accelerometer)

^b^LOA: Limits of agreement calculated using Bland-Altman test

^**c**^Trend, Pearson’s correlation coefficients between the absolute value of the difference versus the average of the two variables (Q1 vs accelerometer). Whether r>0 and p< 0.05, there is heteroscedasticity between the variables

^d^ weighted (quadratic) Cohen’s kappa-coefficient for quartiles comparison

*p ≤.05

**p ≤ .01

## Discussion

The novelty of our study was to examine the reliability and validity of a sedentary behavior questionnaire in South American pediatric population from different cities using harmonized methodology for the first time. The main finding of this study was that the SAYCARE questionnaire showed acceptable reliability for sedentary behavior time in South American children and adolescents. Conversely, the questionnaire had a low validity and systematically underreported sedentary time compared with an accelerometer. Therefore, the SAYCARE questionnaire is a feasible and reliable tool to assess sedentary behavior time in the South American pediatric population, but not valid to estimate sedentary time.

The SAYCARE sedentary behavior questionnaire reported complete information from 83.8% of the participants; whereas, a European multicenter study about this topic reported 75% of complete information [11]. Conversely, we found important decreases in the response rate from Q1 (83.8%) to Q2 (61.8%) surveys. In addition, we found better compliance among participants from private schools and mothers with university degree to the Q2 and accelerometer assessments. We believe that the decrease in responses could be because of decreased participant motivation to complete a second questionnaire within a short lead time. In support of this finding, a comprehensive study involving six Latin American cities showed that socioeconomic status and educational level are limiting factors for education program adherence in adolescence [26]. We believe that this evidence could be extrapolated to our study. However, based on the compliance results of the Q1, we believe that the SAYCARE sedentary behavior questionnaire can be a feasible tool for future association studies in South American children and adolescents.

### Reliability and validity of parent-reported sedentary behavior in children

Findings of the present study indicate that the SAYCARE questionnaire has an acceptable reliability to measure parent-reported sedentary behavior time in the South American children. The reliability performance was probably due to the fact that parents were asked to recall their children’s usual specific behaviors [18, 27]. We also found that reliability was better to identify children who followed the recommendations, than to identify time spent in sedentary behavior. This finding was particularly interesting and can likely be explained by the fact that sedentary behaviors tend to be more stable when compared to active behaviors and therefore, this can facilitate the recall process [28].

Conversely, in our work, there was a low correlation between questionnaires and accelerometers. Our results are not far from previous findings, as a prior systematic review found that the correlation between these methods ranged from − 0.16 to 0.55 [5]. The limited validity of the reported measures compared with the accelerometer was partially explained in the literature: the concomitant and intermittent natures of some sedentary activities are difficult to recall, [1] and metrics (outcome) assessed by questionnaires and accelerometers are “distinct” and “not equivalent” [7]. However, the accelerometer remains the most widely applied instrument and serves as a reference method to validate sedentary behavior questionnaires [5].

Moreover, the SAYCARE sedentary behavior questionnaire showed systematically underreport of sedentary time. The underreporting of sedentary time is not uncommon. Wen et al. showed a mean difference of 175 min/day between an sedentary behavior questionnaire and accelerometer in children [27]. In line with our findings, the authors argue that the subjective tool seems particularly useful to rank children by activity levels, but similarly to other reported tools, the parent-report overestimated time spent in physical activity and underestimate sedentary time compared with the accelerometer [27].

### Reliability and validity of self-reported sedentary behavior in adolescents

In adolescents, despite an acceptable reliability for self-reported sedentary behavior time, the reliability of quartile classification was lower than the acceptable values. We also found no reliability to identify adolescents who followed the recommendations. Evidence suggests that reliability for self-reported information regarding behaviors (e.g., physical activity and sedentary behavior) is lower in young population groups [10]. In addition, sedentary behavior seems not to be settled at young ages and has been characterized as unstructured activities [10]. Moreover, interpretation or understanding of questions is a factor affecting questionnaire reliability and the quality of the reports [10]; memory also plays an important role [29]. The results of the present study are in line with previous systematic reviews that found an acceptable reliability of the questionnaire for time spent in sedentary behavior [3–5].

Our findings indicate that the correlation between self-reported minutes of sedentary behaviors from the SAYCARE and accelerometer-sedentary time was not acceptable. The questionnaire had inadequate ability to identify adolescents according to their sedentary time. Our results are in line with previous systematic reviews [2, 5]. In addition, findings in the present study suggest an important underestimation of the sedentary time compared to accelerometry. Similarly, Affuso et al. found a mean difference of 295 min/day between a self-reported leisure-time sedentary behavior and accelerometer in adolescents [30]. A possible explanation for the higher accelerometer measurements when compared with questionnaires could be the elevated values of absence of movement computed as sedentary time in the accelerometers whereas the questionnaire was developed to measure the time spent on four sedentary activities associated with health outcomes [31, 32].

Thus, studies drawing inferences about total sedentary time (from accelerometers) compared to a set of sedentary behavior (from questionnaires or diaries) should be interpreted with caution. In this sense, we hypothesized that the poor agreement between questionnaire and accelerometer for assessing sedentary behavior found in our study could be likely due to the choice of accelerometers (e.g., Actigraph GT3X) as a reference method rather than the subjective method per se. Although the accelerometer was the most common device used on validity studies, [5, 33] posture sensor (e.g., activPAL) emerges as a new potential reference method [1].

### Strengths and limitations

Our study has some limitations. First, although the sample was robust in size and diversity, the sample was not equally distributed among cities and school types. Second, our sample size was not calculated in separately for children (parent-report) and adolescents (self-report) although the analyzes were done separately. However, in post-hoc analysis the sample size from children (*N* = 55; β < 1%) and adolescents (*N* = 106; β < 1%) remained significant in power. Third, our sample was selected out of convenience. The rationale behind our convenience sample is that the differences between private and public schools across different countries can provide a good idea of the socio-demographic characteristics present in South America [9]. Additionally, although the SAYCARE sedentary behavior questionnaire recorded information about four sedentary activities (including passive playing in children and studying in adolescents), it did not gather all the types of sedentary behaviors that adolescent were able to do [34]. In this sense, to compare with European data, [11, 34] we assessed reported sedentary behaviors, other than screen time, and ranked participants who attend media time recommendations (≤ 120 min/day) [18]. Another concern may regard questionnaire response, for total days in future inferential researches, the sedentary behavior should be restricted to individuals with complete information, due the variation of sedentary time during segments of the week [35]. In the presence of missing data, we strongly encourage the measure of sedentary behavior for week and weekend days in separately. Strengths of this study were a comparison of sedentary behavior questionnaires against an objective measurement (accelerometers) in a multicultural youth group and in low- to middle-income countries, an area of research that has previously been limited [14]; and, the implementation of a harmonized methodology. In according with the literature, this is the first multicenter study addressing comparison between sedentary behavior subjective and objective methodology in South American pediatric population.

## Conclusions

The SAYCARE sedentary behavior questionnaire presents acceptable reliability in South American children and adolescents. However, time spent watching television, using a computer, playing console games, passive playing (only in children) and studying (only in adolescents) is not necessarily indicative of young people’s sedentary time. We recommend the SAYCARE questionnaire as a feasible and reliable tool to assess sedentary behavior time in the South American pediatric population, but not as a proxy of sedentary time.

## Supplementary information

**Additional file 1: Table S1.** Samples composition for the reliability and validity study of SAYCARE sedentary behavior questionnaire.

## Data Availability

The datasets generated and/or analyzed during the current study are not publicly available due confidential term signed up to the conclusion of the study but are available from the corresponding author on reasonable request. The SAYCARE information can be accessed on the website at “http://www.bv.fapesp.br/pt/auxilios/91579/saycare-south-american-youthchild-cardiovascular-and-environmental-study/” and “http://www.bv.fapesp.br/en/auxilios/86359/design-and-implementation-of-the-saycare-study-south-america-youthchild-cardiovascular-and-environ/”

## Abbreviations

Hz: Hertz
k: Kappa agreement coefficient
LOA: Limits of agreement
Q1: Questionnaire first application
Q2: Questionnaire second application
rho: Spearman correlation coefficient
SAYCARE: South American Youth Cardiovascular and Environmental
SB: Sedentary behavior
