# Supplementary material for: Reliability and validity of a sedentary behavior questionnaire for South American pediatric population: SAYCARE study
Source: BMC Med Res Methodol. 2020 Jan 10;20:5. doi: 10.1186/s12874-019-0893-7 (PMC6954524; doi:10.1186/s12874-019-0893-7)
Supplement: Supplementary file 1 — Additional file 1: Table S1. Samples composition for the reliability and validity study of SAYCARE sedentary behavior questionnaire. [file 12874_2019_893_MOESM1_ESM.docx]

**Additional file 1: Table S1.** Samples composition for the reliability and validity study of SAYCARE sedentary behavior questionnaire.

|  | Argentina | Brazil | | Chile | Colombia | Peru | Uruguay | Total |
| --- | --- | --- | --- | --- | --- | --- | --- | --- |
|  | Buenos Aires | Teresina | São Paulo | Santiago | Medellin | Lima | Montevideo |  |
| **Children** |  |  |  |  |  |  |  |  |
| Reliability analysis | n=5 | n=3 | n=11 | n=5 | n=15 | n=8 | n=8 | 55 |
| Validity analysis |  | n=35 | n=48 |  | n=8 | n=2 |  | 93 |
|  |  |  |  |  |  |  |  |  |
| **Adolescents** |  |  |  |  |  |  |  |  |
| Reliability analysis | n=10 |  | n=17 | n=3 | n=44 | n=29 | n=3 | 106 |
| Validity analysis |  | n=26 | n=41 |  | n=12 | n=15 |  | 94 |
|  |  |  |  |  |  |  |  |  |
| **Total** |  |  |  |  |  |  |  |  |
| Reliability analysis | n=15 | n=3 | n=28 | n=8 | n=59 | n=37 | n=11 | 161 |
| Validity analysis |  | n=61 | n=89 |  | n=20 | n=17 |  | 187 |
